# Neuroimaging to Investigate Multisystem Involvement and Provide Biomarkers in Amyotrophic Lateral Sclerosis

**DOI:** 10.1155/2014/467560

**Published:** 2014-04-17

**Authors:** Pierre-François Pradat, Mohamed-Mounir El Mendili

**Affiliations:** ^1^Sorbonne Universités, UPMC Universités Paris 06, UMR 7371, UMR-S 1146, LIB, 75005 Paris, France; ^2^CNRS, UMR 7371, LIB, 75005 Paris, France; ^3^INSERM, UMR-S 1146, LIB, 75005 Paris, France; ^4^Département des Maladies du Système Nerveux, Hôpital Pitié-Salpêtrière (AP-HP), 75005 Paris, France

## Abstract

Neuroimaging allows investigating the extent of neurological systems degeneration in amyotrophic lateral sclerosis (ALS). Advanced MRI methods can detect changes related to the degeneration of upper motor neurons but have also demonstrated the participation of other systems such as the sensory system or basal ganglia, demonstrating *in vivo* that ALS is a multisystem disorder. Structural and functional imaging also allows studying dysfunction of brain areas associated with cognitive signs. From a biomarker perspective, numerous studies using diffusion tensor imaging showed a decrease of fractional anisotropy in the intracranial portion of the corticospinal tract but its diagnostic value at the individual level remains limited. A multiparametric approach will be required to use MRI in the diagnostic workup of ALS. A promising avenue is the new methodological developments of spinal cord imaging that has the advantage to investigate the two motor system components that are involved in ALS, that is, the lower and upper motor neuron. For all neuroimaging modalities, due to the intrinsic heterogeneity of ALS, larger pooled banks of images with standardized image acquisition and analysis procedures are needed. In this paper, we will review the main findings obtained with MRI, PET, SPECT, and nuclear magnetic resonance spectroscopy in ALS.

## 1. Introduction

Amyotrophic lateral sclerosis (ALS) is a neurodegenerative disease characterized by a progressive alteration in the upper, or cortical, motor neurons and lower motor neurons, located in the spinal cord and brainstem. Due to its clinical heterogeneity and the lack of biological markers to diagnose ALS, the delay between the first symptoms and the diagnosis is evaluated at 9–13 months [1]. There is an unmet need to find specific and early biomarkers to help to diagnose and characterize phenotype or progression [2].

Signs of central motor neuron degeneration are often difficult to detect in clinical practice, justifying the interest for objective neuroimaging markers of upper motor neuron (UMN) involvement. It has been estimated that clinical UMN signs are absent at first examination in 7 to 10% of patients who further develop full-blown ALS [3, 4]. The diagnostic delay is increased in patients who present isolated lower motor neuron (LMN) signs [3]. It is needed to rule out other LMN pathologies such as spinal muscular atrophy (SMA), bulbospinal muscular atrophy (Kennedy disease), or multifocal neuropathies with conduction blocks [1]. The apparition of central signs during the follow-up may allow confirming the diagnosis of ALS but this is not the case in all patients, notably because the severity of peripheral signs can mask central signs.

Neuroimaging methods allow investigating* in vivo* the extent of neurological systems degeneration. There is a growing body of evidence demonstrating that ALS is a multisystem neurological disorder. Autopsy studies have shown that degeneration of central nervous system structures is not restricted to the primary motor cortex and the pyramidal tract [5–8]. In addition to motor signs, cognitive signs are detected by neuropsychological tests in about 50% of patients with sporadic ALS and typical frontotemporal dementia (FTD) occurs in approximately 10% of the patients [9, 10]. Association of ALS and FTD occurs in the majority of* C9ORF72*-linked familial ALS (FALS) [11, 12]. Atypical clinical features can be associated, defining “ALS plus” [1] syndromes with signs and symptoms involving the sensory (particularly in* SOD1*-linked FALS) [13–22], extrapyramidal [14, 23–31], cerebellar [23, 32], ocular [33, 34], and autonomic [35] systems.

In this paper, we will review the main findings obtained with different modalities of neuroimaging in ALS. We will especially focus on magnetic resonance imaging (MRI) studies and particularly on spinal cord neuroimaging that has shown great developments in the last few years.

## 2. Magnetic Resonance Imaging

### 2.1. Conventional Magnetic Resonance Imaging

Beyond its role to exclude several “ALS-mimick” syndromes [1], abnormalities suggestive of upper motor neuron involvement are sometimes detected. Several studies, using various modalities (T2*, fluid-attenuated inversion recovery (FLAIR) or fast spin echo proton density-weighted imaging), have shown hyperintensity in the white matter along the corticospinal tract, from the centrum semiovale to the brainstem [36–46]. However, such abnormalities are rare, nonspecific, not readily quantifiable, and do not correlate with disease severity or rate of progression [47]. A cortical atrophy, which is predominant in the frontal region, and a characteristic T2* or FLAIR hypointensity at the level of the primary motor cortex have been described in the literature but are exceptionally detectable in clinical practice [39, 48].

### 2.2. Advanced MRI

Diffusion-based neuroimaging methods allow evaluating the degeneration of white matter fiber bundles. Diffusion results from the random movement of molecules* in vivo*. Diffusion of water in structures such as the cerebrospinal fluid (CSF) or grey matter is isotropic (identical in all directions), whereas in white matter it occurs preferentially along the axis of orientation of the fiber bundles. The application of diffusion gradients in several directions results in diffusion tensor imaging (DTI). An anisotropy map can thus be obtained, which provides information about the microstructural organization of the white matter.

A study published in 1999 demonstrated a decrease in fractional anisotropy (FA) in the intracranial portion of the corticospinal tract (subcortical white matter, internal capsule, and brainstem) [49]. It has been confirmed by numerous other studies [50–60]. Abnormalities have been observed in patients who had no UMN signs at the time of MRI investigation but developed pyramidal tract symptoms later in the course of their disease, suggesting that DTI could contribute to earlier diagnosis of ALS in patients with pure LMN involvement [51]. It has been suggested that a decreased fractional anisotropy (FA) in the corpus callosum (CC) was a good DTI marker in patients with ALS [61]. These changes may correspond to degeneration of transcallosal fibers passing between primary motor cortices. Another study showed that FA in motor-related regions of the CC is more affected than in other CC areas in ALS patients [62].

Tractography is a method based on DTI that enables reconstruction of the three-dimensional geometry of the pyramidal tract [63] and allows establishing an FA profile along the pyramidal tract [64]. A tractography study showed that the decreases in FA are largely limited to the precentral areas in patients with ALS [52]. Using tractography to segment the corticobulbar tract, lower FA was measured in patients with bulbar-onset versus limb-onset disease [65].

**Figure 1:**
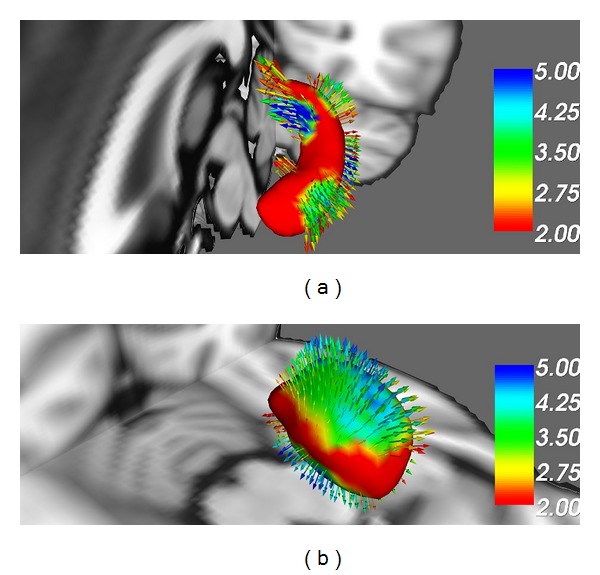
Evidence of hippocampal and basal ganglia involvement in amyotrophic lateral sclerosis. Comparative surface-based vertex analyses between healthy controls and* C9orf72* hexanucleotide repeat negative ALS patients corrected for age and multiple comparisons reveal significant left hippocampal (a) and thalamic changes (b) (Courtesy of Peter Bede-Trinity College Dublin).

By using a voxel-by-voxel approach, which consists of comparing groups of patients without an* a priori *predefined region of interest, our team and other groups have showed that abnormalities on DTI were detectable outside the primary motor regions, thereby confirming that ALS is a multisystem degenerative disorder [53]. Diffuse lesions have also been observed using voxel-based morphometry (VBM), a technic allowing automated segmentation and quantification of grey and white matter volumes to study regional differences [66]. Recently authors used high-resolution T1-weighted imaging data for model-based subcortical registration and segmentation to explore the involvement of subcortical structures [67]. They used vertex-wise statistics that provide quantitative, visual, surface-projected information about the shape of the various subcortical structures. This analysis revealed changes affecting the basal ganglia including the superior and inferior aspects of the bilateral thalami, the lateral and inferior portion of the left hippocampus, and the medial and superior aspect of the left caudate (Figure 1). The authors conclude that dysfunction of frontostriatal networks is likely to contribute to the unique neuropsychological profile of ALS, dominated by executive dysfunction, apathy, and deficits in social cognition.

The discovery that the presence of a hexanucleotide expansion in* C9orf72 gene *[11, 12] was associated with FTD in ALS led to specific neuroimaging investigations. A recent study used tract-based spatial statistics of multiple white matter diffusion parameters, cortical thickness measurements, and VBM analyses in* C9orf72*-negative patients with ALS carrying the* C9orf72* hexanucleotide repeat expansion [68]. The result is that extensive cortical and subcortical frontotemporal involvement was identified in association with the* C9orf72* genotype, compared to the relatively limited extramotor pathology in patients with* C9orf72*-negative ALS [67]. In contrast, a DTI study showed that patients with* SOD1 *gene-linked FALS showed less extensive pathologic white matter in motor and extramotor pathways compared with patients with sporadic ALS [69].

In clinical practice, the diagnostic value of FA measurement remains limited mainly because of the overlap between the values measured in ALS patients and those in control subjects. In one study, measurement of FA in the internal capsule to detect central motor neuron lesions compared with healthy subjects had a sensitivity of 95%, but the specificity was only 71%, with a positive predictive value of 82% [46]. A recent individual patient data (IPD) meta-analysis using corticospinal tract data suggested that the diagnostic accuracy of DTI lacks sufficient discrimination [59]. Of 30 identified studies, 11 corresponding authors provided IPD and 221 ALS patients and 187 healthy control subjects were available for the study. The pooled sensitivity was 0.68 (95% CI: 0.62–0.75), and the pooled specificity was 0.73 (95% CI: 0.66–0.80).

### 2.3. Spinal Cord Imaging

Spinal MRI has the advantage to investigate the two motor system components that are involved in ALS, that is, the lower and upper motor neuron. Although widely applied to the brain, advanced methods such as DTI are challenging at the spinal level because of (i) the small size of the cord relative to the brain (~1 cm diameter in the human) requiring higher spatial resolution and thus decreasing the signal-to-noise ratio, (ii) physiological motions (respiration, cardiac) that may bias anisotropic diffusion coefficient estimation and create ghosting artifacts [70–72], (iii) partial volume effects that are more problematic in the cord due to the surrounding cerebrospinal fluid [73], (iv) chemical-shift artifacts arising from the epidural fat and other nearby structures, and (v) geometric distortions arising from magnetic field inhomogeneities in nearby intervertebral disks and lungs. The latter point is particularly challenging in diffusion MRI since usual sequences based on echo planar imaging (EPI) are very sensitive to such artifacts [74, 75]. In past years, with the development of new methods, such as cardiac and respiratory gating [75–78], it has been shown that DTI and magnetization transfer (MT) imaging were feasible to detect changes in the spinal cord in ALS [79–81]. In a recent study, we have shown that abnormalities in the spinal cord using a multiparametric MRI approach combing DTI, MT ratio (MTR), and atrophy measurements correlated with functional impairment [79]. In this study, local spinal cord atrophy was correlated with muscle deficits and with the motor evoked potential amplitude measured by transcranial magnetic stimulation (TMS), an index of LMN dysfunction. It suggests that regional atrophy is a sensitive biomarker of motor neuron loss in the anterior horns of the spinal cord.

Conversely, DTI and MTR changes in the corticospinal tract correlated with the higher facilitation motor threshold measured by TMS, a parameter that reflects the functionality of the pyramidal tract. Interestingly, changes of DTI metrics demonstrated a subclinical involvement of sensory pathways.

Recently, new acquisition strategies have been proposed to overcome the inherent difficulties in diffusion-weighted imaging of the spinal cord. A promising high-resolution DWI sequence (Syngo RESOLVE) has been proposed [82]. The RESOLVE sequence allows minimization of susceptibility distortions and T2* blurring. Furthermore, it can be combined with other acquisition strategies such as reduction field-of-view (rFOV) [83–85] and parallel imaging [86] to provide fiber tractography in large portions of the spinal cord. Such advances open doors to an accurate quantification of DTI metrics profile along the corticospinal tract or sensory tracts. This is fundamentally needed to clarify some physiopathological aspects of ALS disease, for instance, the dying-back versus dying-forward hypotheses and the possible sensory afferents involvement [79]. In parallel, the new generation of 3 T MRI scanners equipped with 300 mT/m gradients [87, 88] provides new exploratory dimensions for white matter microstructures in the spinal cord [89, 90]. As showed* in vivo* as well as* ex vivo* for the brain, the new MRI scanners improved tissues sensitivity, signal-to-noise ratio (SNR), and spatial and angular diffusion resolution in practical time.

**Figure 2:**
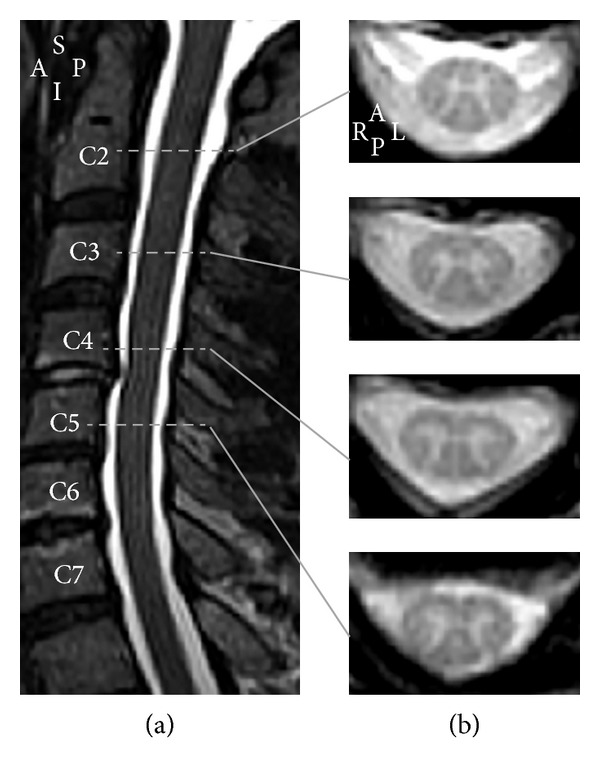
(a) T2-weighted turbo spin echo midsagittal section in a healthy subject (male, 62 years old), showing the anatomical landmarks of the cervical spinal cord. (b) T2*-weighted 2D gradient recalled echo axial sections at the vertebral levels C2, C3, C4, and C5 for the same subject (voxel  size = 0.7 × 0.7 × 3 mm). Images have been acquired using a 3 T MRI system (TIM Trio 32-channel, Siemens Healthcare, Erlangen, Germany). A: anterior; I: inferior; L: left; P: posterior; R: right; S: superior.

MRI pathological spinal cord studies were mainly focused on white matter integrity where grey matter was not for a great interest until the past few years. This was mainly related to the difficulties in imaging the spinal cord due to low SNR as well as contrastdifference between CSF and white and grey matter at 1.5 T. Passing to higher magnetic field strength (3 T and 7 T) [91–93], the construction of adapted coils for spinal cord imaging [94–96] and the adaptation of existing sequences [91, 97] bring new horizons for spinal cord anatomical explorations. Three recent studies showed the feasibility of white/grey matter imaging and presented reliable tools for anatomical structures characterization in controls [98, 99] and multiple sclerosis patients [100] using 3 T MRI-systems. In parallel, one study has showed preliminary results at 3 T of the construction of a probabilistic atlas and anatomical template of the human cervical and thoracic spinal cord that included CSF and white and gray matter [97]. Such atlas can be used for atrophy localization in ALS patients using VBM by adapting the methodology proposed in [101] to the case of 3 T MR images (Figure 2).

Such advances bring new perspectives for neurodegenerative diseases and particularly for ALS. Quantifications of white and grey matter degeneration are currently feasible. MRI sequences, image processing, and statistical tools have been already developed [91, 97, 101–103] and are almost ready for use to figure out some of the mechanisms involved in spinal cord tissues degeneration in ALS.

### 2.4. Functional MRI

Functional MRI (fMRI), by measuring cortical blood oxygen level dependent (BOLD) signal changes, provides a tool to study cortical function and reorganization. Regional modifications in cerebral blood flow have been studied during hand motor tasks [104]. Several studies have demonstrated that cerebral activation involved more extensive cortical regions than in control subjects. However, whether it reflects cortical reorganization [104]or it is the result of cortical functional adaptation due to peripheral weakness [105] remains a matter of debate. Using a simple hand motor task when the motor deficit is still moderate, a study showed that cerebral activation is correlated with the rate of disease progression suggesting that brain functional rearrangement in ALS may have prognostic implications [106].

Unlike the traditional fMRI, the recently developed resting-state functional (rfMRI) techniques avoid potential performance since rfMRI does not require the subjects to perform any task [107, 108]. Studies that have been carried out in ALS provide abundant evidence for a reorganisation of various cerebral networks [108–113]. Interestingly, one study showed that rfMRI changes correlated with the rate of disease progression and duration [114].

## 3. Nuclear Magnetic Resonance Spectroscopy

Nuclear magnetic resonance spectroscopy (NMRS) allows measuring the neurochemical profile of a particular region of the brain* in vivo*. The main peak is N-acetylaspartate (NAA), which is considered as a marker of neuronal integrity. Several studies have demonstrated a decrease in NAA [115, 116] and/or ratios of NAA with choline-containing compounds (Cho) and creatine (Cr) [NAA/Cho and NAA/Cr ratios] at the level of the motor cortex of patients with ALS [115, 117].

For diagnosis applications, studies suggested that NMRS spectroscopy may discriminate between patients with classical ALS and patients with progressive muscular atrophy, a disease confined to LMNs [118, 119]. The diagnostic value is limited because of an overlap between those values in ALS patients and healthy controls. It has been suggested that the combination of N-acetylaspartate (NAA) and myo-inositol may improve the specificity of the test but further studies are needed [120]. Using a whole-brain resonance spectroscopic imaging approach, NAA showed a significant relationship with disability [121]. A cross-sectional study assessing proton NMRS of the cervical spine showed that NAA/Cr and NAA/Myo ratios were reduced in patients with ALS but also in* SOD1*-positive people at risk for FALS, suggesting that neurometabolic changes occur early in the course of the disease process [122]. Recent advances in high resolution NMRS at 3 T allow direct quantification of GABA in the cortex [123]. Recently, a study in a small number of subjects showed that decreased levels of GABA were present in the motor cortex of ALS patients compared to healthy controls [124]. It suggests that a loss of inhibition by interneurons may play a role in neurodegeneration through excitotoxicity mechanisms.

## 4. Positron Emission Tomography

Positron emission tomography (PET) and monophotonic emission tomography (single photon emission computerized tomography (SPECT)) are nuclear imaging techniques which use various tracers to either reveal neuron dysfunction or investigate a pathogenic mechanism involved in the disease. Using SPECT, several studies have demonstrated a decrease in cerebral blood flow after injection of hexamethyl-propyleneamine oxime labelled with technetium-99 m (99 mTc). They showed abnormalities in the primary motor cortex [125–128], which can also extend in an anterior fashion into the frontal lobes, particularly in patients with associated cognitive problems [129]. Studies using PET with 2-fluoro-2-deoxy-glucose also showed a variable decrease in cerebral glucose metabolism at rest [130–132]. Interestingly, a PET study found not only hypometabolic but also hypermetabolic areas in the brain of sporadic ALS patients, possibly due to increased FDG uptake by astrocytes and/or microglia [133]. In a recent study, the authors report an FDG metabolism study in patients with the* C9orf72* mutation compared to nonmutated ALS patients (either with or without dementia) [134]. The conclusion is that* C9orf72* mutated ALS patients t have a more widespread central nervous system involvement than ALS patients without genetic mutations, with or without dementia.

A PET study showed a widespread loss of binding of the GABAA ligand [^11^C]-flumazenil in sporadic ALS patients [135]. Because GABAA receptors are widely distributed in the cerebral cortex and are located both on pyramidal cells and interneurons, [^11^C]-flumazenil provides a means of detecting motor and extramotor dysfunction in ALS. It was shown that abnormalities were less extensive in patients with* SOD1*-linked familial ALS patients, suggesting that GABA-ergic neurotransmission may be less severely impaired in these cases [136]. Nigrostriatal dysfunction has also been shown by both PET [137] and SPECT studies [138].

PET imaging can also detect inflammatory processes that are implicated in the pathogenesis of ALS. Assessment of microglial activation can be performed through neuroimaging of the 18 kDA translocator protein (TSPO) that is present in activated glial cells, using selective TSPO-selective radioligands such as ^11^C-PK11195. Extensive cortical abnormalities in fixation of this tracer, predominantly in the frontal and temporal lobes have been observed in one study [139]. The DPA714 radioligand has a longer half-life, a better bioavailability, and less nonspecific binding than PK11195 [140]. This allows the examination of milder microglial activation by PET. A prospective study showed significant binding of DPA714 both in motor cortex areas and also in temporal areas since the earlier stages of the disease [141]. With the continuing development in new radioligands, PET imaging may potentially provide tools to monitor the effect of drugs targeting inflammation.

## 5. Conclusion 

In the recent years, thanks to technological and methodological developments, neuroimaging was revealed as an indispensable research tool to understand the pathophysiology of ALS. Because ALS is a multisystem disorder and not a pure motor neuron disease, there is a need to investigate* in vivo* the participation of other systems such as the sensory or extrapyramidal systems. Neuroimaging studies have already provided insights about the potential role of sensory feedback, inflammation, and loss of inhibition by interneurons in the pathogenesis of ALS. Studies on presymptomatic carriers of mutations responsible for FALS provide the unique opportunity to study the very early mechanism that triggers the neurodegenerative cascade leading to the loss of motor neurons.

Among MRI techniques, although DTI initially appeared as the most promising diagnostic tool, the disappointing results of a meta-analysis suggest that a multiparametric approach will be required to make neuroimaging a critical component in the workup of ALS. Development of spinal cord imaging will be a key element to provide useful biomarkers as sensitive and specific as possible to help diagnosis and to characterize phenotype or progression. Due to the intrinsic heterogeneity of ALS, larger pooled banks of images with standardized image acquisition and analysis procedures are needed. For this purpose, the NeuroImaging Society in ALS (NISALS) has emerged in 2010 and has an interactive web Platform (http://nedigs05.nedig.uni-jena.de/nisals/) to provide quality controlled MRI data for the international scientific community.
